# Dietary and physical activity patterns related to cardio-metabolic health among Malaysian adolescents: a systematic review

**DOI:** 10.1186/s12889-019-6557-z

**Published:** 2019-02-28

**Authors:** Shooka Mohammadi, Muhammad Yazid Jalaludin, Tin Tin Su, Maznah Dahlui, Mohd Nahar Azmi Mohamed, Hazreen Abdul Majid

**Affiliations:** 1 Department of Social and Preventive Medicine, Faculty of Medicine, University of Malaya, 50603 Kuala Lumpur, Malaysia; 2 Department of Paediatrics, Faculty of Medicine, University of Malaya, 50603 Kuala Lumpur, Malaysia; 3 Department of Sports Medicine, Faculty of Medicine, University of Malaya, 50603 Kuala Lumpur, Malaysia; 4 South East Asia Community Observatory (SEACO), Jeffrey Cheah School of Medicine and Health Sciences, Monash University Malaysia, 47500 Bandar Sunway, Malaysia; 5 Faculty of Public Health, Universitas Airlangga, 60115 Jawa Timur, Indonesia; 6 Department of Nutrition, Harvard T.H. Chan School of Public Health, Harvard University, Boston, 02115 MA USA

**Keywords:** Dietary patterns, Cardio-metabolic, Physical activity, Malaysian adolescents, Systematic review

## Abstract

**Background:**

A sedentary lifestyle and an unhealthy diet are major factors in the increasing prevalence of obesity among Malaysian adolescents. The purpose of this systematic review is to compile the evidence from observational and intervention studies among Malaysian adolescents to evaluate the associations between diet and physical activity (PA) as determinants of cardio-metabolic risk factors.

**Methods:**

A systematic search of Medline via the PubMed, Science Direct, Cochrane Review and Web of Science databases was conducted for studies on the associations between diet and PA factors and cardio-metabolic risk factors among Malaysian adolescents aged 13–18 years that were published until 31 August 2017. The search results were independently screened and extracted by two reviewers.

**Results:**

From over 2,410 references retrieved, 20 full texts articles were screened as potentially relevant. Seventeen (16 cross-sectional and one intervention) met the inclusion criteria for data extraction and analysis. All 17 studies were rated as poor quality and the majority had made insufficient adjustment for confounders. As regards the effect of diet and PA on cardio-metabolic health, the intakes of energy (*n* = 4) and macronutrients (*n* = 3) and meal frequency (*n* = 5) were the most commonly studied dietary factors, while the PA score and level were the most commonly studied PA factors. In addition, BMI and body weight were the most common cardio-metabolic health outcomes. The studies showed that obese and overweight adolescents consume significantly more energy and macronutrients. They are also more likely to skip their daily meals compared to their normal weight peers. In most studies, the direction of the PA effect on body weight was unclear. Some studies found that higher PA is associated with a lower risk of overweight and obesity. However, the associations are often small or inconsistent, with few studies controlling for confounding factors.

**Conclusions:**

This review identified a lack of evidence and well-conducted prospective studies on the effect of diet and PA on cardio-metabolic health of Malaysian adolescents.

**Electronic supplementary material:**

The online version of this article (10.1186/s12889-019-6557-z) contains supplementary material, which is available to authorized users.

## Background

Non-communicable diseases (NCDs) are the most common cause of death in Malaysia [1]. Specifically, cardio-metabolic diseases (e.g. coronary heart disease, stroke and type 2 diabetes) contributed towards half of the mortality rate in 2011, and among the adult population, 2.6 million (15.2%) had diabetes, 5.8 million (35.1%) had hypertension and 6.2 million (32.7%) had hypercholesterolemia [2]. Moreover, being overweight or obese during adolescence has been found to have both immediate and long-term negative impacts on health such as increased risk of cardiovascular disease (CVD), diabetes and cancer [3–6], as well as metabolic disorders (e.g. increased blood cholesterol and glucose levels, hypertension and insulin resistance) [7–9].

The National Health and Morbidity Survey 2017 (NHMS) [10] and several recent studies [11–13] have reported an increasing trend in the prevalence of obesity among Malaysian adolescents. Furthermore, it has been reported, Malaysia has the second highest prevalence of overweight or obesity in this age group (23.7%) in the Southeast Asia region [14]. The underlying causes of NCDs in Malaysia have been attributed to rapid nutritional and socio-cultural transitions. Hence, positive changes in individual health-related behaviours such as diet and physical activity (PA) are likely to be major factors in the prevention of obesity and CVD [15].

Unhealthy foods and dietary patterns, as well as a lack of PA and increased sedentary behaviour (SB) have been associated with obesity and cardio-metabolic risk factors [16–18], although the list of factors that have robust causality is short. For instance, a recent systematic review of studies around the world found the link between unhealthy dietary patterns and cardio-metabolic changes in adolescents and children [19]. The aim of that review was to determine how much a food pattern categorized as ‘unhealthy’ could have the impact on the inflammatory and biochemical markers in the populations under study. The patterns were classified as unhealthy when correlated with the intake of food that were ultra-processed, lacked fibre and had high sodium, fat and refined carbohydrate content [19]. More specifically, several studies found that Western dietary patterns, which contain high-sugar and high-fat food, are related to greater obesity risk in adolescents, while healthier dietary patterns characterized by a high intake of vegetables, whole grains and legumes may decrease the overweight/obesity risk [20–22].

There is a large body of evidence in the systematic reviews that suggest less sedentary time is associated with lower cardio-metabolic risk in adolescents [23, 24]. In addition, higher amounts of moderate to vigorous physical activity (MVPA) have been associated with better cardio-metabolic health in adolescents, regardless of the amount of sedentary time [25, 26]. Even low-intensity PA has been shown to be favourably associated with cardio-metabolic biomarkers [26]. Also, evidence-based studies have revealed that PA has favourable effects on adiposity and non-traditional cardiovascular risk factors (inflammatory markers and irregular heart rate levels) in adolescents with normal body weight, plasma lipid and lipoprotein levels and blood pressure (BP) [27, 28].

In Malaysia, findings from a cohort study showed that there is a low level of PA among the majority of adolescents (64%) [29], as well as inadequate nutrient intake [30, 31] and insufficient healthy dietary patterns [10]. In addition, it has been reported that 50.1% of Malaysian students spend at least 3 hours in a typical or usual day in sitting activities [10].

Thus, understanding how specific diet and PA behaviours affect health in Malaysia could inform intervention targets to decrease the cardio-metabolic risk among adolescents. However, existing systematic reviews of studies that investigated the associations between lifestyle factors and cardio-metabolic health revealed a dearth of studies in Malaysian populations. Therefore, the aim of this systematic review is to summarize the evidence reported by observational and interventional studies that have been conducted on the lifestyle (nutrition and PA) of Malaysian adolescents in secondary schools (13–18 years old) thus this will assist to understand the associations between diet and PA and cardio-metabolic risk factors in this age group. It is hoped that the findings of this systematic review will be useful in providing an indication of the interventions needed to improve the dietary and PA patterns and consequently the cardio-metabolic health of Malaysian adolescents.

## Methods

The review protocol registered at PROSPERO (registration number: CRD42017074556) and this systematic review was conducted in accordance with the PRISMA guidelines.

### Literature search

Observational and intervention studies published up to 31 August 2017 were identified through a structured search of the Medline via PubMed, Science Direct, Cochrane Review and Web of Science databases. The search strategy focused on three key concepts: population (i.e. Malaysian adolescents, secondary school), behaviour (i.e. healthy lifestyle, healthy eating and PA) and cardio-metabolic health outcome. The complete list of search strategies are presented in Additional file 1. Two reviewers (SM and ZT) independently screened the results against inclusion criteria then they extracted the selected full text articles independently. Out of the 2,327 titles screened by the two reviewers, 2,307 (99%) were excluded by both, 20 (1%) were included by both and the exclusion of a further three articles on which there were divergent decisions was resolved after discussion.

### Selection of studies

This review included all observational (cross-sectional or prospective) studies that examined the associations between diet and PA factors and cardio-metabolic health outcome, as well as all interventional studies that aimed to reduce the cardio-metabolic risks (through healthy lifestyle approaches) among Malaysian adolescents. After removing duplicates, studies were selected in accordance with the following inclusion criteria: (i) interventional studies aimed at decreasing the cardio-metabolic risks among Malaysian adolescents via a change in diet and PA; (ii) observational studies that reported associations between diet or PA behaviour and cardio-metabolic risk factors; (iii) involving healthy Malaysian adolescents, aged between 13 and 18 years; (iv) and fully published. The exclusion criteria were: (i) studies that focused on unhealthy adolescents without comparison, e.g., obese only; (ii) papers that not peer-reviewed or published abstracts at the scientific conference; and (iii) not in humans. A full-text screening was performed by two reviewers (SM, ZT) who selected the articles based on the inclusion criteria. Disagreements between the reviewers were resolved through discussion with LJ and HM.

### Quality assessment

Risk of bias was assessed using the modified Newcastle–Ottawa Scale for cross-sectional studies [32] and intervention study. Quality assessment was done independently by two of the reviewers (SM and ZT) on four of the included studies then they completed the quality assessment of the remaining studies, independently. Where there were disagreements, the two reviewers discussed the issues until consensus was achieved.

### Data extraction and synthesis

Two independent reviewers (SM and ZT) extracted the data on study characteristics (author, geographic location and sample size), participant characteristics (age, ethnicity, urbanity, maternal education and household income), diet/PA assessment method, specific diet/activity factors, cardio-metabolic health outcome and covariates. The cardio-metabolic health related factors included body mass index (BMI), waist circumference (WC), blood pressure (BP), total cholesterol level including total high-density lipoprotein (HDL) cholesterol and low-density lipoprotein (LDL).

The level of significance for associations was set at *p* < 0.05. For studies that applied univariate and multivariate analyses, only the multivariate results were considered in this review. In order to ease the interpretation of the findings, conceptually similar factors were combined.

## Results

### Search results

**Fig. 1:**
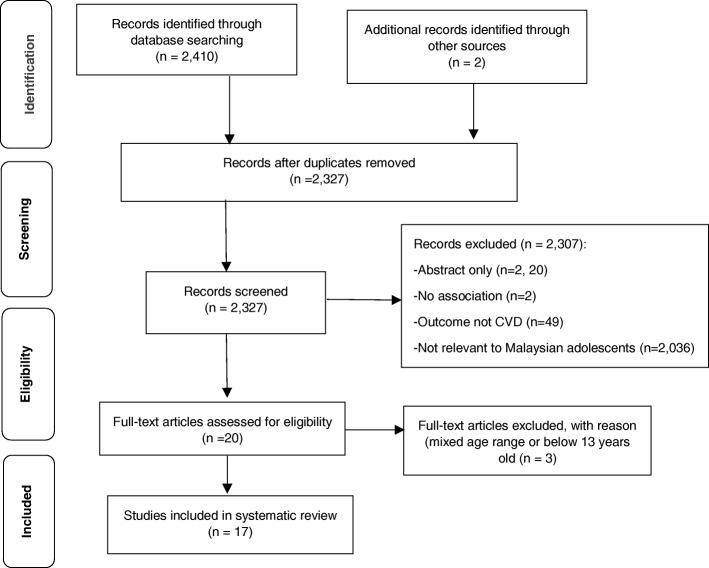
Flow Diagram of study selection

The initial multi-database search yielded 2,410 publications. Figure 1 illustrates the screening and selection process. After excluding duplicates, 2,327 records were screened. Of these, 2,307 were excluded mainly because they were not relevant to Malaysian adolescents (*n* = 2,036) or they only consisted of an abstract (*n* = 220).

Table 1 provides details of the design and methodology of each of the 17 included articles (16 cross-sectional studies (29-31, 33-45) and one intervention study [46]. Most studies included both boys and girls (*n* = 14), multiple ethnicities, i.e. Malay, Indian and Chinese (*n* = 13) and were conducted in Central Malaysia and Kuala Lumpur (*n* = 8). The sample sizes of the studies ranged from 81 to 40,011 adolescents.

**Table 1:** Characteristics of the included studies

Author, year	Setting/Urbanity	Sample	Age (y) Mean ± SD	Ethnicity	Maternal education	Income (RM)	Diet measure	PA measure	Cardio metabolic health outcome	Covariates
Zalilah et al. (2006) [38]	NR	618 (♂ ♀)	13.1 ± 0.7	Malay, Chinese Indian	NR	(Mean ± SD) ♂ RM 1,002 ± 940 ♀ RM 1,082 ± 1,123	3DD	3DD	Body weight status	Ethnicity, household income
Majid et al.(2016) [30]^a^	Kuala Lumpur, ,Selangor, Perak/ Urban &rural	794 (♂ ♀)	12.9 ± 0.3	Malay, Chinese Indian, Other	Secondary :66%	49 % low SES	7DH		BMI (kg/m^2^)	NR
Loh et al. (2017) [33]	Kuala Lumpur /Urban	873 (♂ ♀)	13^a^	Malay, Chinese Indian, Other	Secondary:61.4 %	NR	OQ (CNQ)		WC, TG, HDL LDL, SB DB. FBG Insulin	Sex, ethnicity, maternal education, PGS, physical activity, BMI, child nutrition questionnaire scores
Nurul-Fadhilah et al.(2013) [34]	Kota Bharu in Kelantan/Urban	236 (♂ ♀)	15.3 ± 1.9	Malay	NR	(Mean ± SD) RM 2,191±2,553	FFQ		BMI (z score), WC, Body Fat (%), Body weight	Age, household income, PGS, eating out, snacking, energy intake, PA
Chew et al. (2016) [40]	Hulu Langat in Selangor/ NR	832 (♂ ♀)	Median(IQR) 16 (15,16)	Malay, Chinese Indian, Other	Secondary: 59%	RM 2,001-5,000 (41%)	FFQ	IPAQ	Abdominal obesity	Sex, ethnicity, BMI
Pon et al. (2004) [39]	Teluk Intan in Perak/ Rural	100 (♀)	14.8 ± 1.2	Malay, Chinese, Indian	NW: primary:44% OW: secondary 48%	NW: RM 501-1000 (40%) OW: < RM 500 (36%)	FFQ/ 1*24R		Body weight status	NR
Chin & Nasir (2009) [35]	Kuatan in Pahang/ / NR	407 (♀)	15.3 ± 1.9	Malay, Chinese Indian, Other	Secondary:57.0 %	(Mean ± SD) RM 3,266 ± 2,566	OQ (EBQ)		Body weight status	NR
Rezali et al. (2012) [42]	Kajang In Selangor/ Urban	382 (♂ ♀)	14.0 ± 0.8	Malay, Chinese Indian	NR	NR	2DD	2DD	BMI (z-score), Body weight status	Physical activity, body image
Boon et al. (2012) [43]	Kuala Lumpur/ Urban	156 (♂ ♀)	14.1 ± 0.8	Malay, Chinese, Indian	NR	RM 2,000-5,999 (57%)	1*24R		BMI (kg/m2),body weight status	NR
Cheah et al. (2011) [41]	Kuching in Sarawak/ NR	316 (♂ ♀)	Range: 13-17	Malay, Chinese Indian, Other	Secondary: 63.9 %	(Mean ± SD) RM 3,653 ± 3,740	FFQ	Media time	BMI (z-score)	NR
Cynthia et al.2013 [44]	Puchong in Selangor/ Urban	408 (♂ ♀)	13.2 ± 0.3	Malay, Chinese Indian, Other	Secondary: 56 %	41.9% < RM 3999	OQ (PESQ)		Body weight status BMI (kgm2)	Sex, ethnicity, household income, total energy intake
Marzuki et al.(1991) [46]	NR/ NR	110 (♂)	Range: 16-17	Malay	NR	NR	1*24R		TG, TC, HDL, LDL	NR
Baharudin et al. (2014) [36]	NR / NR	40011 (♂ ♀)	13.4 ± 2.2	NR	NR	NR		PAQ-C	BMI (z-score)	Age, sex, BMI, breakfast intake,school session
Su et al. (2014) [29]^a^	Kuala Lumpur, Selangor, Perak/ Urban &rural	1327 (♂ ♀)	12.9 ± 0.3	Malay, Chinese, Indian, Other	NR	NR		PAQ-C	BMI (kg/m2), WC, Body fat (%)	Age, sex, ethnicity, place of residence
Teo et al. (2014) [37]	Kota Bharu in Kelantan/ NR	454 (♂ ♀)	15.3 ± 1.9	Malay, Chinese	NR	NR		PAQ-C	Weight status, waist circumference, % body fat	Age, PGS, ethnicity, SES, total energy intakes, fat density intakes , total SB levels (h/day)
Farah Wahida et al.(2011) [31]	Kuantan in Pahang/ NR	360 (♂ ♀)	13.2 ± 0.3	Malay, Chinese, Indian, Other	50.6 % Secondary	NR		PAQ-C	BMI	NR
Dan et al. (2011) [45]	Kuantan in Pahang/ NR	400 (♂ ♀)	13.23 ± 0.31	Malay, Chinese Indian	12.29 ± 3.39 (Mean± SD)years of education	38.5 %> RM 3000		PAQ-C	BMI (z-score)	NR

*Abbreviations*: ♂ Male, ♀ Female, *SES* Socioeconomic status, *NR* Not reported, *RM* Malaysian ringgit (currency), *BMI* Body Mass Index, *FFQ* Food frequency questionnaire, X*24R 24 hour recall completed over X days, *XDH* X days diet history, *OQ* Oher Questionnaire, *EBQ* Eating Behaviours Questionnaire, *CNQ* Child Nutrition Questionnaire, *PGS* pubertal growth status, *SB* Sedentary behaviour, *PA* Physical Activity, *PAQ-C* Physical Activity Questionnaire for Children, *HDL* High-density lipoprotein, *LDL* Low-density lipoprotein, *WC* Waist Circumference, *BP* blood pressure, *NW* Normal weight, *OW* Overweight

^a^MyHeART study

In the included studies, diet was mostly measured with food frequency questionnaires (*n* = 4), multiple-day food diaries (*n* = 2) and 24-h dietary recall (*n* = 3), while PA was mostly self-reported via the Physical Activity Questionnaire for Children (PAQ-C) questionnaire (*n* = 5). Only nine studies reported information on adolescents’ maternal education and household income and in all of those studies the majority of the participants had mothers who had completed a secondary school education and were in households with a moderate household income. Sex and ethnicity were the most common adjusted covariates; however, only nine studies provided adjusted covariates. Overall, all 17 studies were rated as poor quality. The primary reasons for poor quality were that the statistical analyses did not adjust for confounders, e.g., age, sex, sample size did not justify, etc.

### Diet and cardio-metabolic health

Table 2 summarizes the associations between potential diet determinants and cardio-metabolic health. These determinants were grouped into three categories: energy and nutrients, foods, and eating frequency. Energy intake (*n* = 4), macronutrients (*n* = 3) and meal frequency (*n* = 5) were the most commonly studied dietary correlates of cardio-metabolic health. Furthermore, BMI and body weight were the most common outcomes of cardio-metabolic health.

**Table 2:** Summary of association between health-related determinants of diet and cardio metabolic health outcome

	Author, year	Cardio metabolic health outcome	Association	*P*-value
Energy and nutrients				
Energy intake	Zalilah et al. (2006) [38]	Body weight status	UW vs. NW vs. OW (Mean ± SE) ♂ 2198±92 vs. 2133±77 vs. 2262±84 ♀ 1916±74 vs. 1903±57 vs. 2138±57	p = NS p < 0.05
	Rezali et al. (2012) [42]	BMI (z-score)	Beta=0.001	p < 0.01
	Majid et al.(2016) [30]	BMI (kg/m^2^)	NW vs. UW vs. OW vs. Obese (Mean (95% CI) 1673.6 (1642.2–1705.0) vs. 1571.9 (1530.1–1613.7) vs. 1742 (1657.7–1826.5) vs. 1987 (1825.8–2149.3)	P < 0.001
	Pon et al. (2004) [39]	Body weight status	OW vs. NW (Mean ± SD) 1518 ± 572 vs. 1636 ± 528	p=NS
Carbohydrates	Rezali et al. (2012) [42]	Body weight status	UW vs. NW vs. OW (Mean ± SD) 198±69 vs. 197±64 vs. 211±65	p=NS
	Zalilah et al. (2006) [38]	Body weight status	UW vs. NW vs. OW (Mean ± SE) ♂ 281±2 vs. 271±10 vs. 285±11 ♀ 247±10 vs. 252±8 vs. 272±8	p=NS p<0.05
	Majid et al. (2016) [30]	BMI (kg/m^2^)	NW vs. UW vs. OW vs. Obese (Mean (95% CI) 231.2 (226.3–236.1) vs. 218.5 (211.9–225.2) vs. 235.4 (224.1–246.8) vs. 269.7 (246.7–292.7)	p<0.001
Protein	Rezali et al. (2012) [42]	Body weight status	UW vs. NW vs. OW (Mean ± SD) 60±24 vs. 62±19 vs. 67±20	p = NS
	Zalilah et al. (2006) [38]	Body weight status	UW vs. NW vs. OW (Mean ± SE) ♂ 89±6 vs. 80±5 vs. 86±5 ♀ 72±3 vs. 70±2 vs. 80±3	p =NS p<0.05
	Majid et al. (2016) [30]	BMI (kg/m^2^)	NW vs. UW vs. OW vs. Obese mean (95% CI) 62.4 (60.9–63.8) vs. 56.9 (55.1–58.7) vs. 64.2 (60.4–67.9) vs.(62.5–78.5) vs. 70.5 ( 62.5–78.5)	p < 0.001
Fat	Rezali et al. (2012) [42]	Body weight status	UW vs. NW vs. OW (Mean ± SD) 61 ± 23 vs. 66 ± 22 vs. 72 ± 22	NS
	Zalilah et al. (2006) [38]	Body weight status	UW vs. NW vs. OW (Mean ± SE) ♂ 80 ± 4 vs. 81±3 vs. 86 ± 4 ♀ 70 ± 4 vs. 69 ± 3 vs. 81± 37	p =NS p<0.05
	Majid et al.(2016) [30]	BMI (kg/m^2^)	NW vs. UW vs. OW vs. Obese (Mean (95% CI) 55.7 (54.4–57.0) vs. 52.0 (50.1–54.0) vs. 60.6 (56.7–64.5) vs. 70.3 (63.4–77.0)	p < 0.001
Cholesterol	Majid et al.(2016) [30]	BMI (kg/m^2^)	NW vs. UW vs. OW vs. Obese (mean (95% CI) 225.2 (216.5–233.8) vs. 212.1 (200.8–223.4) vs. 234.7 (209.9–259.5) vs. 262.5 (209.2–315.8)	p = NS
Saturated fatty acid	Majid et al. (2016) [30]	BMI (kg/m^2^)	NW vs. UW vs. OW vs. Obese (mean (95% CI) 10.9 (10.4–11.3) vs. 10.4 (9.8–11.0) vs. 11.9 (10.2–13.6) vs.13.1 (11.6–14.6)	p = NS
Unsaturated fatty acid	Majid et al. (2016) [30]	BMI (kg/m^2^)	Unsaturated fatty acid NW vs. UW vs. OW vs. Obese (Mean (95% CI) 8.4 (8.1–8.8) vs.7.8 (7.4–8.2) vs. 8.3 (7.6–9.0) vs.9.9 (8.1–11.7)	p = NS
			Polyunsaturated fatty acid NW vs. UW vs. OW vs. Obese (Mean (95% CI) 6.1 (5.9–6.4) vs. 5.7 (5.4–6.0) vs. 6.3 (5.8–6.9) vs. 7.8 (6.3–9.3)	p = NS
Saturated (palm olein) vs. Polyunsaturated (soybean oil) cooking oils	Marzuki et al.(1991) [46]	Triglycerides (mmol/L)	Baseline vs. Palm oil diet vs. Soy bean diet (Mean ± SE) 0.85 ± 0.04 vs. 0.74 ± 0.03 vs.1.09 ± 0.06	p < 0.001
		Total chlesterol (mmol/L)	Baseline vs. Palm oil diet vs. Soy bean diet (Mean ± SE) 3.89±0.09 vs. 3.86 ± 0.08 vs.3.95 ± 0.09	p = NS
		HDL cholesterol (mmol/ L)	Baseline vs. Palm oil diet vs. Soy bean diet (Mean ± SE) 1.39± 0.03 vs. 1.33± 0.03 vs. 1.32± 0.03	p = NS
		LDL cholesterol (mmol/ L)	Baseline vs. Palm oil diet vs. Soy bean diet (Mean ± SE) 2.37±0.09 vs. 2.37 ±0.08 vs. 2.41± 0.09	p = NS
Sugar	Majid et al. (2016) [30]	BMI (kg/m^2^)	NW vs. UW vs. OW vs. Obese (Mean (95% CI) 33.8 (32.3–35.3) vs. 33.6 (31.6–35.6) vs 35.3 (30.8–39.9) vs. 48.9 (39.0–58.7)	p < 0.001
Fiber	Majid et al. (2016) [30]	BMI (kg/m^2^)	NW vs. UW vs. OW vs. Obese (Mean (95% CI) 2.9 (2.8–3.1) vs.2.8 (2.6–2.9) vs. 3.0 (2.7–3.4) vs4.1 (2.9–5.4)	p = NS
	Chew et al. (2016) [40]	Abdominal obesity	Normal vs. WC vs. AO (%) Daily: 35.6 vs. 38.3 Weekly: 38 vs. 34.0 Rarely: 26.4 vs. 27.7	p = 0.479
Foods				
Sugar sweetened beverages	Loh et al. (2017) [33]	WC (cm)	r2(95% CI) r2= 0.67 (69.1–72.5)	p < 0.001
		Triglycerides (mmol/ L)	r2(95% CI) r2=0.1 (0.13–1.27)	p< 0.01
		HDL cholesterol (mmol/ L)	r2(95% CI) r2=0.19 (0.99–2.29)	p < 0.001
		LDL cholesterol (mmol/ L)	r2(95% CI) r2=0.12 (2.04–4.31)	p < 0.001
		Systolic BP (mmHg)	r2(95% CI) r2=0.31 (100–107)	p < 0.001
		Diastolic BP (mmHg)	r2(95% CI) r2=0.16 (59–64)	p < 0.001
		Fasting blood glucose (mmol L)	r2(95% CI) r2=0.03 (4.51–5.36)	p < 0.001
		Insulin (uUmL)	r2(95% CI) r2=0.42 (12.54–16.21)	p < 0.001
	Chew et al. (2016) [40]	Abdominal obesity	Higher SSB frequency (OR=0.974, 95% CI 0.891, 1.064)	p = 0.557
	Cheah et al. (2011) [41]	BMI (z-score)	NW vs. OW (%) Every day: 4 vs. 12 Seldom: 40 vs. 52	p = 0.06
Legumes	Chew et al. (2016) [40]	Abdominal obesity	Higher legume frequency (OR=1.102 95% CI 0.934, 1.105)	p = 0.717
Vegetarian diet	Chew et al. (2016) [40]	Abdominal obesity	Vegetarians vs. non-vegetarians (OR=2.512, 95% CI 0.293, 21.526)	p = 0.401
Eating frequency				
Meal frequency	Chew et al. (2016) [40]	Abdominal obesity	Irregular vs. regular meals (OR=3.193, 95% CI 1.043, 9.774)	p = 0.042
	Pon et al. (2004) [39]	Body weight status	Regular Mealtime NW vs. OW (%) Yes: 32 vs. 40 Sometimes: 42 vs. 34 No: 26 vs. 26	p = NS
	Boon et al. (2012) [43]	BMI (kg/m2)	3M+3S vs. 3M+2S vs. 3M+1S vs. 3M vs. <2M,2,3 S vs. <2M+0,1S (Mean ± SD) 19 ± 3.3 vs. 20.3 ± 4.6 vs. 19.5± 4.3 vs. 18.5 ± 3.2 vs. 18.9 ±3.7 vs. 20.5 ± 3.6	p > 0.05
		Body weight status	3M+3S vs. 3M+2S vs. 3M+1S vs. 3M vs. <2M,2,3 S vs. <2M+0,1S (%) UW : 16 vs.16.7 vs. 12.1 vs.25 vs. 13.6 vs. 8.3 NW 72 vs. 52.8 vs.69.7 vs.62.5 vs.81.8 vs.70.8 OW 8 vs. 22.2 vs. 12.1 vs. 12.5 vs. 0 vs. 20.8 Obese 4 vs. 8.3 vs. 6.1 vs. 0. 4.5 vs. 0	p > 0.05
	Chew et al. (2016) [40]	Abdominal obesity	Not daily vs. daily dinner (OR=0.516, 95% CI 0.179-1.488)	p = 0.221
	Nurul-Fadhilah et al.(2013) [34]	BMI (z-score)	Breakfast ≥5 times/week vs. <5 times/week (Mean ± SE) ♂- 0.19 ± 0.15 vs. 0.40 ± 0.19 ♀ -0.39 ± 0.17 vs. 0.21 ± 0.18	p = 0.021 p = 0.017
		WC (cm)	Breakfast ≥5 times/week vs. <5 times/week (Mean ± SE) ♂ 66.3 ± 1.3 vs. 70.6 ±1.6 ♀ 62.8 ± 1.2 vs. 67.6 ± 1.3	p = 0.006 p = 0.008
		Body weight (Kg)	Breakfast ≥5 times/week vs. <5 times/week (Mean ± SE) ♂50.0 ± 1.5 vs. 56.4 ± 1.9 ♀46.3 ± 1.5 vs. 51.0 ± 1.6	p = 0.010 p = 0.039
		Body Fat (%)	Breakfast ≥5 times/week vs. <5 times/week (Mean ± SE) ♂ 15.1±1.2 vs. 20.4±1.5 ♀ 30.4±0.9 vs. 33.0±1.0	p = 0.006 p = 0.045
Meal frequency	Chew et al. (2016) [40]	Abdominal obesity	Normal WC vs. AO (%) Breakfast Daily: 48.9 vs. 44.1 Not daily : 51.1 vs. 55.9	p = 0.380
	Chew et al. (2016) [40]	Abdominal obesity	Normal WC vs. AO (%) Lunch Daily : 84.1 vs. 83 Not daily: 15.9 vs. 17.0	p = 0.771
	Chew et al. (2016) [40]	Abdominal obesity	Not daily vs. daily dinner (OR=0.516, 95% CI: 0.179, 1.488)	p = 0.221
	Pon et al. (2004) [39]	Body weight status	NW vs. OW (%) Dinner daily Yes: 12 vs.. 10 Dinner sometimes : 42 vs. 52 No: 46 vs. 38	p = NS
	Pon et al. (2004) [39]	Body weight status	Skipping Daily Meals NW vs. OW (%) Yes: 40 vs. 16 Sometimes: 48 vs. 54 No: 12 vs. 30	p < 0.05
	Chin & Nasir (2009) [35]	Body weight status	UW vs. NW vs. OW (%) Never skip 45.8 vs. 37.8 vs. 25.0 Skipped at least one meal: 50 vs. , 53.1 vs. 51.3 Skipped all 3 meals daily: 4.2 vs. 9.1 vs.23.7	p < 0.05
Snacks frequency	Chew et al. (2016) [40]	Abdominal obesity	Snacking Between Meals Normal WC vs. AO (%) Daily: 28.8 vs. 22.3 Weekly: 40.8 vs. 46.8 Rarely: 30.3 vs. 30.9	p = 0.371
	Cheah et al. (2011) [41]	BMI (z-score)	Cereal-snacking frequency NW vs. OW (% respondents) Everyday: 27 vs. 32 Seldom: 40 vs. 20	p = 0.146
	Cheah et al. (2011) [41]	BMI (z-score)	Junk food- snacking frequency NW vs. OW (% respondents) Everyday: 12 vs. 4 Seldom: 32 vs. 44	p = 0.212
	Cheah et al. (2011) [41]	BMI (z-score)	NW vs. OW (% respondents) Everyday: 8 vs. 0 Seldom: 39 vs. 59	p = 0.294
	Chew et al. (2016) [40]	Abdominal obesity	Fast food-snacking frequency Normal WC vs. AO (%) Daily: 4.6 vs. 1.1 Weekly: 44.3 vs. 41.5 Rarely: 51.1 vs. 57.4	p = 0.193
	Pon et al. (2004) [39]	Body weight status	Snacking Between Meals NW vs. OW (%) Yes: 58 vs. 64 No: 42 vs .36	p = NS
Frequency of family meals away from home	Cynthia et al.2013 [44]	Body weight status	0-2 times vs. 3-6 times vs.> 7 times (Mean ± SE) 50.09 ± 0.92 vs.47.26 ± 1.07 vs.49.94 ± 1.37	p = 0.881
		BMI (kgm2)	0-2 times vs. 3-6 times vs.> 7 times (Mean ± SE) 19.75 ± 0.30 vs. 18.80 ± 0.35 vs. 19.84 ± 0.45	p = 0.933

*Abbreviations*: ♂ Male, ♀ Female, *UW* Underweight, *NW* Normal weight, *OW* Overweight, *BMI* Body Mass Index, *AO* abdominal obesity, *WC* waist circumference, *SE* standard error, *(3M+3S)* 3 meals + 3 snacks, *(3M+2S)* 3 meals + 2 snacks, *(3M+1S)* 3 meals + one snack, *(3M)* 3 meals, *(≤2M±2,3S)* meal skippers consumed snacks frequently, *(≤2M±0,1S)* meal skippers consumed snacks only one time or never, *NS* not statistically significant (p> 0.05), *SE* standard error

#### Energy and nutrients

The dietary factors that have been studied in relation to cardio-metabolic health included intakes of energy, carbohydrates, protein, fat, sugar, fibre and dietary cholesterol.

##### Energy

Four cross-sectional studies investigated the association between energy intake and cardio-metabolic health, all of which focused on body weight [30, 42] or BMI (38, 39) as the outcome. In the three studies with unadjusted associations, overweight and obese adolescents had significantly higher mean energy intakes compared to normal-weight adolescents [30, 38], while no difference was observed in the mean energy between normal and underweight adolescents [39]. Only one study adjusted for covariates (i.e., PA and body image) and showed a small but significant association between energy intake and BMI (β = 0.001, *p* < 0.05) [42].

##### Macronutrients

Three cross-sectional studies [30, 38, 42] examined the association between macronutrients (i.e., carbohydrates, protein, total fat, unsaturated and saturated fat) and body weight and BMI, while one intervention study [46] evaluated the effect of saturated vs. polyunsaturated fat consumption on triglycerides and total, HDL and LDL cholesterol. All these studies reported unadjusted mean intakes of macronutrients and two showed significantly higher intakes of carbohydrates, protein and total fat in obese vs. overweight and normal-weight adolescents [30] or in girls only [38]. No association was observed between saturated and polyunsaturated fat intake and cardio-metabolic outcomes. However, in one intervention study, triglyceride levels increased after consuming a diet high in soybean oil (polyunsaturated fat) compared to a diet high in palm oil (saturated fat) for 5 weeks (1.09 vs. 0.74 mmol/L, *p* < 0.001) [46].

##### Other nutrients

Two cross-sectional studies [30, 40] examined the associations between fibre, BMI and abdominal obesity and one study investigated the relationship between dietary cholesterol, sugar and BMI [46]. However, a significant association was found only for sugar and it was indicated that obese adolescents consumed significantly more sugar when compared to normal-weight adolescents [46].

#### Foods

The foods that have been studied in relation to cardio-metabolic health included sugar-sweetened beverages (SSB), legumes and the vegetarian diet.

##### Sugar-sweetened beverages

Three studies considered the association between SSB consumption and cardio-metabolic health [33, 40, 41]. No association was found between SSB consumption and abdominal obesity [40] and between SSB consumption and BMI (z-score) [41]. Only one study reported that SSB intake was deleteriously associated with increased metabolic parameters (i.e. HDL cholesterol, LDL cholesterol, triglycerides, systolic BP, diastolic BP and WC) when adjusted for confounders (i.e., sex, ethnicity, maternal education, pubertal stage, PA, BMI, child nutrition questionnaire scores) [33].

##### Legume intake

One study looked at the association between legume intake and abdominal obesity but did not find any association [40].

##### Vegetarian diet

One study investigated the relationship between following a vegetarian diet and abdominal obesity. After controlling for various confounders (i.e., sex, ethnicity and BMI), no association was found between being a vegetarian and abdominal obesity [40].

#### Eating frequency

Eating frequency has been studied in relation to cardio-metabolic health by considering three correlates: meal frequency and snacking frequency and frequency of family meals away from home (FMAFH).

##### Meal frequency

Five cross-sectional studies investigated associations between dietary patterns and cardio-metabolic health [34, 35, 39, 40, 43]. Only two studies reported adjusted associations [34, 40]. One study showed an adjusted association between irregular meal frequency and abdominal obesity (odds ratio (OR) = 3.193, 95% confidence interval (CI) 1.043-9.774; *p* = 0.042)) after controlling for various confounders (e.g., sex, ethnicity and BMI, etc.) [40]. In the other study with adjusted associations, frequent breakfast intake was found to be significantly related to lower levels of BMI, WC, body weight and body fat (BF) (%) after adjusting for various confounders (i.e., age, daily energy intakes, frequency of eating out, snacking practices, household income, pubertal growth status and daily PA levels) [34]. However, two other studies reported unadjusted associations between skipping daily meals and body weight and showed that more overweight adolescents skipped one or more daily meals as compared to their normal-weight counterparts [35, 39]. However, there was no association between body weight or BMI and meal patterns, snacking patterns [39, 43] or frequency of meals and dinner [39]. In addition, no association was found between meal frequency or snacking between meals and having abdominal obesity [40].

##### Snacking frequency

Three studies investigated the association between frequency of snacking and cardio-metabolic health including abdominal obesity [40], BMI (z-score) [41] and body weight [39], but none found significant associations.

##### Frequency of family meals away from home

One cross-sectional study examined the association between FMAFH in the last 7 days and cardio-metabolic risk. However, body weight and BMI were not significantly different by FMAFH categories after adjusting for covariates, i.e., sex, ethnicity, household income and total energy intake [44].

### Physical activity and cardio-metabolic health

Table 3 summarizes the associations between PA determinants and cardio-metabolic health. The PA score, level of PA, and sedentary behaviour (SB) were explored in ten studies. In addition, BMI and weight status were the most common cardio-metabolic health outcomes in these studies.

**Table 3:** Summary of association between health-related determinants of PA and cardio metabolic health outcome

	Author, year	Cardio metabolic health outcome	Association	*P*-value
Physical activity	Chew et al. (2016) [40]	Abdominal obesity	Normal WC vs.AO 4170 ± 4122 vs. 4021 ± 4199	p = 0.666
	Rezali et al. (2012) [42]	Body weight status	UW vs. NW vs. OW &Obese (Mean ± SD) 1.06 ± 0.31 vs. 1.27 ± 0.31 vs. 1.58 ± 0.33	p < 0.01
		BMI (z-score)	Non OW vs. OW & obese Beta=5.34	p < 0.05
	Su et al. (2014) [29]	BMI (kg/m2)	OR=−0.058	p < 0.05
		WC (cm)	OR=−0.069	p <0.05
		Body fat (%)	OR=−0.088	p < 0.05
	Zalilah et al. (2006) [38]	Body weight status	UW vs. NW vs. OW (Mean ± SE) ♀ 490 ± 17.7 vs. 559 ± 13.6 vs. 689 ± 13.6 ♂715 ± 28.7 vs. 763 ± 3.9 vs. 1,059 ± 26.1	p < 0.001 p < 0.001
	Farah Wahida et al. (2011) [31]	BMI (kg/m^2^)	r=-0.03	p = NS
	Teo et al. (2014) [37]	Weight status	Low (<1.5 h) vs. High (≥1.5 h)(ref) OR(95%CI) ♂ OR = 3.0 (1.1 – 8.1) ♀ OR = 1.7( 0.6 – 5.0)	p = 0.029 p = 0.302
	Teo et al. (2014) [37]	Waist circumference (cm)	Low (<1.5 h) vs. High (≥1.5 h)(Mean, 95%, CI) ♂ 69.7 (67.4 - 71.9) vs. 67.9 (65.7-70.0) ♀ 65.7 (64.4- 67.0) vs. 64.6 (62.4-66.7)	p = 0.263 p = 0.370
	Teo et al. (2014) [37]	Body Fat (%)	Low (<1.5 h)vs High (≥1.5 h)( mean, 95%, CI) ♂ 20.0 (18.2 - 21.9) vs. 15.9 (14.2-17.7) ♀ 32.6 (31.5 - 33.6) vs. 31.3 (29.6 - 33.0)	p = 0.002 p =0.219
	Baharudin et al. (2014) [36]	BMI (z-score)	Physical inactivity NW (ref) UW(OR=1.2, 95% CI 1.06 -1.31) OW (OR=1.1 , 95% CI 0.99 -1.18) Obese(OR=1.2 ,95% CI 1.11-1.37)	p = 0.003 p < 0.077 p < 0.001
Physical activity intensity	Teo et al. (2014) [37]	Weight status	Low (<1 h) vs. High (≥1 h) (ref) OR(95%CI) ♂ OR=3.8 (1.4–10.1) ♀ OR=2.3 (0.7–7.8)	p = 0.008 p = 0.198
	Teo et al. (2014) [37]	Waist circumference (cm)	Low (<1 h) vs. High (≥1 h) ( Mean, 95%, CI) ♂ 70.2 (67.8- 72.6) vs. 67.6 (65.6- 69.7) ♀ 65.9 (64.6- 67.1) vs. 63.5 (61.1- 66.0)	p= 0.120 p=0.103
	Teo et al. (2014) [37]	Body Fat (%)	Low (<1 h) vs. High (≥1 h) (Mean, 95%, CI) ♂ 20.7 (18.7-22.6) vs. 15.8 (14.2-17.5) ♀32.5 (31.5-33.5) vs. 31.2 (29.2-33.2)	p =0.0001 p = 0.267
	Chew et al. (2016) [40]	Abdominal obesity	Normal WC vs.AO High 54% vs. 52.3% Moderate 31% vs. 30.2% Low 12.9% vs. 17.4%	p = 0.492
	Rezali et al. (2012) [42]	Body weight status	Non-OW vs. OW & obese (%) Sedentary: 93.5 vs. 6.5 Light: 70.1 vs. 29.9 Moderately/vigorously: 38.9 vs. 61.1	p < 0.01
	Zalilah et al. (2006) [38]	Body weight status	(light) UW vs. NW vs. OW (Mean ± SE) ♀1,156 ± 9.4 vs. 1,147 ± 7.2 vs. 1,148 ± 7.2 ♂1,163 ± 9.4) vs. 1,180 ± 7.8 vs. 1,157 ± 8.6	p = NS p = NS
			(Moderate) UW vs. NW vs. OW (Mean ± SE) ♀ 245±8.2 vs. 252 ±6.3 vs. 251±6.3 ♂ 207± 8.0 vs. 205±6.7 vs. 218 ±7.3	p = NS p = NS
			(High) UW vs. NW vs. OW (Mean ± SE) ♀ 40 ± 3.7 vs. 41 ± 2.8 vs. 41±2.8 ♂ 69 ± 4.8 vs. 55 ± 4.0 vs. 65±4.4	p = NS p = NS
	Dan et al. (2011) [45]	BMI (z-score)	Low vs. Moderate/High n (%) UW: 10 (34.5) vs.19 (65.5) NW: 100 (35.7) vs. 180 (64.3) OW 32 (35.2) vs. 59 (64.8) r=-0.043	p = NS
Sedentary behaviour	Teo et al. (2014) [37]	Weight status	High (<3.5 h) vs. Low (≥3.5 (ref) OR (95%CI) ♂ 2.4 (0.9–6.3) ♀ 2.8 (1.0–7.5)	p = 0.06 p = 0.04
	Cheah et al. (2011) [41]	BMI (z-score)	Normal vs. High 10.364 ± 5.44 vs. 10.66 ± 5.34	p = 0.729

*Abbreviations*: ♂ Male, ♀ Female, *UW* underweight, *NW* Normal weight, *OW* overweight, *BMI* Body Mass Index, *AO* abdominal obesity, *WC* waist circumference, *SE* standard error, *NS* not statistically significant (p> 0.05)

#### Physical activity score

Seven studies investigated the association between PA and cardio-metabolic health [29, 31, 36, 37, 38, 40, 42). Three of them did not find any significant associations between PA and BMI [31], abdominal obesity [40] or WC [37]. However, significant associations were found between PA and weight status [37, 38, 42], BMI [29, 36, 42], the percentage of body fat [29, 37] and WC [29] in the other four studies.

Furthermore, in two studies the mean PA score for overweight and obese adolescents was significantly higher compared to that for underweight and normal-weight respondents [38, 42]. In addition, PA was found to make a significant contribution to overweight and obesity in adolescents after adjusting for body image and energy intake (R2 = 0.213, β= 5.346, *p* < 0.01) [42].

One study explored the association between physical inactivity and BMI [36]. After controlling for other factors (i.e., age, sex, BMI, breakfast intake, school session), the study found that adolescents who perceived themselves as overweight (AOR (adjusted odds ratio) = 0.8; 95% CI: 0.76–0.89) or obese (AOR = 0.9; 95% CI = 0.78–0.94) had lower odds of being physically inactive compared to those who perceived their body weight as normal [36].

One cross-sectional study examined the associations between PA duration, body weight status and body fat percentage after adjustment for age (in years), ethnicity, household income, pubertal Tanner stage, total daily energy and fat intakes and total SB levels (h/day). While no significant difference was found in girls [37], the study reported that adolescent boys with daily total PA levels of < 1.5 h/day had a significantly higher risk of being obese (OR 3.0; 95% CI: 1.1–8.1; *p* < 0.05) than boys with greater daily total PA levels. Although there was no comparable association between the PA measures and obesity risk in adolescent girls, boys with low PA duration (< 1.5 h a day) had significantly higher percentage of BF [37]. This is in line with another study that demonstrated that high PA scores were associated with decreased WC and BMI [29].

#### Physical activity intensity

Five cross-sectional studies evaluated the association between PA intensity and cardio-metabolic health [37, 38, 40, 42, 45]. Overall, the evidence on the relationship between PA and weight status was equivocal. Three of the studies did not show evidence of an association between PA intensity and BMI (z-score) [45], body weight status [38] or abdominal obesity [40]. However, significant associations between PA intensity and weight status were reported in two studies (37, 42,) and between PA intensity and BF percentage in one study [37].

It was also reported that overweight and obese adolescents were more involved in an MVPA lifestyle compared to underweight and normal-weight adolescents (χ2 = 39.056, *p* < 0.01) [42]. Another study highlighted that adolescent boys whose daily MVPA intensity was less than 1 hour had a four times higher risk of being obese (OR 3.8; 95% CI: 1.4–10.1; *p* < 0.01) after adjusting for confounders (i.e., age, pubertal Tanner status, ethnicity, household income, total energy intake, total fat density intake and total SB levels). However, the same study found that there was no association between PA intensity measures and obesity risk in adolescent girls [37]. Also, boys with low MVPA were more likely to have a higher %BF compared to boys in the high MVPA group, after full adjustment for these confounders and daily SB duration. No such difference was found for the intensity of daily PA in girls [37].

#### Sedentary behaviour

Two studies evaluated the association between screen-based sedentary practices and BMI (z-score) [41] and weight status [37, 41]. One of the studies did not find an association [41], whereas the other found a negative association between daily sedentary practices and obesity risks in girls [37]. A significant, three times greater probability of risk of being obese was determined among girls with SB levels ≥ 3.5 h/day than in girls with SB levels of < 3.5 h/day (OR 2.8; 95% CI: 1.0–7.5; *p* < 0.05), after adjustment for confounders (i.e. age, pubertal Tanner status, ethnicity, household income, total PA levels, total energy and fat density intakes [37].

## Discussion

This review analysed the evidence presented in 17 studies regarding the associations between diet and PA behaviours and cardio-metabolic health among Malaysian adolescents. The results of this systematic review found weak to moderate evidence to support association between sedentary lifestyle and unhealthy eating patterns with cardio-metabolic risk. There were some conflicting evidence related to the relationship between certain dietary and PA factors with cardio-metabolic health in Malaysian adolescents. While a number of potential determinants have been studied in the Malaysian context, this review clearly shows that for many variables, the evidence is lacking due to the scarcity of studies. Also, among the studies that do exist, the associations are often small or inconsistent, with few studies controlling for confounding factors.

In addition, the majority of studies thus far have focused on body weight and BMI as the outcomes related to cardio-metabolic health, but have not provided sufficient evidence on other related cardio-metabolic health outcomes such as lipid profile and BP. In the studies selected for this review, body fat was estimated by using a number of approaches, such as BMI (kg/m^2^, z-score) or % body fat. This insufficient standardization in the methods applied, as well as hampering comparisons decreases the precision of the information in the studies [47].

All the studies were rated as poor quality owing to insufficient adjustment for confounders in the data analyses and lack of justification for the sample sizes. While there is consistent evidence for an association between eating frequency, PA and PA duration and cardio-metabolic health, there is limited evidence on the effect of all other factors, i.e., sugar, SSB and SB, due to the scarcity of studies or due to multiple studies reporting conflicting evidence in respect of the effects of PA intensity, energy and macronutrients.

Eleven out of the 17 selected articles found a significant association between the intakes of energy and macronutrients, sugar, consumption of SSB and meal frequency and the cardio-metabolic health of adolescents. This systematic review identified that obese and overweight adolescents have significantly higher intakes of energy and macronutrients compared to normal-weight adolescents [30, 42] or girls only [38]. However, it has also been pointed out in one review that there is conflicting evidence regarding the association between intake of total energy and obesity among children, which may be described by insufficient control of possible covariates such as parental overweight and underreporting [48]. In addition, a variety of tools used to assess energy and macronutrient intakes in the reviewed studies may not have been robust enough to capture information for this group. Findings from a previous systematic review suggest that 24-h dietary recall and the dietary history interview are the most precise tools for children aged 4–14 years, where the parent or both the child and parent are the reporters [49].

Regardless of the importance of undertaking studies on dietary patterns, only a few such studies have been conducted among Malaysian adolescents, so there is very little evidence on the association between diet and cardio-metabolic health. This systematic review also identified that, in both sexes, frequent breakfast eaters had significantly lower body weight, WC, BMI z-score, and total BF% [34]. In addition, significant associations were reported between irregular meal frequency and abdominal obesity [40] and between skipping daily meals and body weight in which overweight adolescents skipped one or more daily meals as compared to their normal-weight counterparts [35, 39]. It has been explained in other studies that breakfast skippers tend to consume high energy-dense foods and have an increased tendency of overeating at other meals during the day [50, 51].

In agreement with the results identified in this review, a previous systematic review found that there was little or inconsistent evidence to support a correlation between being overweight and skipping breakfast, eating away from home, daily eating frequency, intake of large food portions, irregular meals, eating until full, snacking, eating quickly and consumption of fast food [52]. A recent systematic review also highlighted that an unhealthy dietary pattern may have an impact on the cardio-metabolic risks among adolescents, and considering the small number and limitations of the included studies, further researches should be undertaken to strengthen the evidence on this association [16].

Only one cross-sectional study in this systematic review revealed a significant association between SSB consumption and higher levels of cardio-metabolic risk in Malaysian adolescents [33]. The study showed that average SSB consumption among Malaysian adolescents was 177.5 mL/day, which was lower than the European adolescents (227.7 mL/day) [53]. Given the rising paediatric obesity rates in Malaysia, an underestimation of SSB intake is possible [54]. However, a strong association has been identified in two systematic reviews and in a meta-analysis on the independent role of SSB consumption in weight gain and obesity in children and adolescents [55, 56]. The meta-analysis revealed that a one serving per day raise in SSB was related with a 0.06-unit increase in BMI over a 1-year period among children and adolescents [56]. One possible explanation for this association could be that the excessive sugars consumed may be stored as fat, leading to weight gain and increased adiposity [57].

It was pointed out in the intervention study included in this systematic review that triglyceride levels increased after consuming a diet high in soybean oil (polyunsaturated fat) compared to a diet high in palm oil (saturated fat) for 5 weeks [46]. However, there was weak evidence that there were no inverse impacts of palm olein as a cooking oil on the plasma lipid profiles of Malaysian adolescents [46]. Also, the study used a small sample size (110) and only investigated male adolescents, thus it is difficult to generalize the results and the evidence overall was weak. In contrast, a systematic review of 51 studies revealed that both acceptable and unacceptable alterations in CVD risk markers were observed when primary dietary fats were substituted by palm oil, whereas only favourable changes happened when trans-fatty acids were substituted by palm oil [58].

This systematic review also investigated the effect of objectively measured PA patterns on cardio-metabolic health in adolescents. However, the imprecise measurement of PA and small sample sizes, as well as the lack of studies, weakened the observed relationships. In this systematic review, the mean PA score [36, 38, 42], PA intensity [42], PA intensity in boys [37] were identified as significantly higher than underweight and normal-weight respondents. However, in another cross-sectional study higher daily total PA scores and duration were associated with decreased obesity risk [37] in male adolescents and reduced WC and BMI [29]. Furthermore, the same study found a significant association between daily sedentary time and obesity risk in girls [37]. In contrast, it has been reported in two systematic reviews that PA levels were lower in overweight compared to normal-weight adolescents [59, 60]; only one cross-sectional study included in this systematic review was in agreement with this result but supplied weak evidence [29]. It has been stated in another systematic review that association between SB and adiposity in adolescents was small to very small and there was little to no evidence that this association was causal [61]. All the included studies related to PA in this systematic review were cross-sectional which, as highlighted in another systematic review, means that it is not possible to draw proper conclusions on cause and effect [62].

### Limitations

This systematic review is the first to summarize the findings on the effects of the associated determinants of diet and PA on cardio-metabolic health among adolescents in Malaysia. Although the reviewed studies found significant associations between diet and PA and cardio-metabolic health, some studies found no significant relationship. The inconsistency of these findings can be related to the inconsistency in the methods used to assess diet and body fat, as well as to the use of incorrect techniques [63].

In addition, all the included articles except one were cross-sectional and used invalid or poorly validated PA and diet measurement tools as well as self-reported data. Beside the differences in the methods adopted to evaluate the exposure and outcome variables, other factors related to the lack of consistency in the results of these studies are: a) various adjustment variables were used in the statistical analyses, which can impact directly on the significance of the associations; b) the wide age variation in the adolescent populations studied, which can affect the way of measuring exposure and outcome along with the degrees of error in information and measurement. Furthermore, inadequate confounder control also appears to be a problem in the majority of studies. For instance, in some studies, the analyses were not adjusted for relevant socio-demographic factors such as sex, age and/or socioeconomic status (SES).

The inability to find positive associations between intakes of macronutrients and cardio-metabolic health has been highlighted in a previous systematic review [64], in which it was determined that predicting true dietary intake was often difficult because underreporting may happen when the information is forgotten or deliberately left out. This imprecision makes it hard to analyse energy and macro- and micronutrient intakes, along with their associations with cardio-metabolic changes [19].

Also, the incapability to determine positive associations between some risk factors and unhealthy foods in cross-sectional studies may be partly described by alterations in eating habits or dietary limitations when body composition changes already occur in adolescents, such as overweight and obesity, known as reverse causality [65, 66]. In addition, the constant use of BMI to assess adiposity is uncertain since it is a method based only on body weight and does not differentiate lean mass from fat mass. Therefore, the method can incorrectly categorize an individual as thin, when in reality, they have a high amount of body fat, or on the other hand, it can consider an individual with a large quantity of lean body mass as overweight/obese [47, 67]. An earlier systematic review highlighted that BMI is often used a proxy for obesity, but using it to assess body composition (e.g. percentage body fat) or body fat distribution (e.g. WC or waist-hip ratio) may lead to incorrect categorization [63].

Furthermore, the studies reviewed here only used questionnaires; they did not use an accelerometer or other related electronic devices. However, a previous systematic review concluded that questionnaires that have both acceptable reliability and validity are not currently available for PA assessment in adolescents [68]. Hence, in light of the above, the main limitation of this systematic review is the low quality of all included studies.

This review also investigated the link between physical activity patterns and cardio-metabolic risk factors in adolescents. To date, most reviews that have investigated PA patterns have not determined which specific activity patterns of activity may be essential for health because their primary attention has been on total volumes of PA and/or SB [62].

In addition, many studies included samples that were non-representative or only representative of a limited geographical area. The majority of studies focused on specific states such as Kuala Lumpur, Selangor and Pahang, whereas only a few were conducted in the northeast regions of Malaysia. This indicates that scientific research related to diet/PA and cardio-metabolic health in Malaysian adolescents has yet to spread to all regions in the country. Furthermore, the included studies were heterogeneous in terms of conceptualization, measurement, sample and analyses, and therefore it was not possible to evaluate the overall strength of any identified associations. However, improving the quality of further studies could lead to more consistency among studies and greater sureness in the identified correlates and determinants of diet and PA.

### Future research directions

To get a better understanding of the impact of factors associated with obesity-inducing behaviours in adolescents, longitudinal intervention studies to assess body fat through more precise methods should be performed in which valid or objective measurement tools are used to focus on health-related PA and dietary patterns among Malaysian adolescents. Since the incidence of obesity and associated comorbidities is rising in this population, a more robust intervention should be planned with the aim of decreasing short- and long-term health damage and future healthcare costs.

## Conclusion

This review draws consideration to the methods used for evaluating diet and PA in studies conducted in Malaysia, whose heterogeneity hampers reliability as well as comparability. This review found insufficient evidence on the association between diet, PA and cardio-metabolic health in Malaysian adolescents. However, the results of this systematic review suggest that the intake of unhealthy foods (higher energy density and higher amount of macronutrients) and less PA appear to be related to higher cardio-metabolic risks in adolescence.

## Additional file

Additional file 1: Search terms. This table gives the search terms used for PubMed, Science Direct, Cochrane Review and Web of Science. (DOCX 29 kb)

## Abbreviations

AO: Abdominal obesity
BMI: Body Mass Index
BP: Blood pressure
CI: Confidence interval
CVD: Cardiovascular disease
FFQ: Food frequency questionnaire
HDL: High-density lipoprotein
LDL: Low-density lipoprotein
MVPA: Moderate to vigorous physical activity
NS: Not statistically significant
NW: Normal weight
OR: Odd ratio
OW: Overweight
PA: Physical activity
PAQ-C: Physical activity questionnaire for Children
RM: Malaysian ringgit (currency)
SB: Sedentary behaviour
SE: Standard error
SES: Socioeconomic status
UW: Underweight
WC: Waist circumference
