# Supplementary material for: Dietary and physical activity patterns related to cardio-metabolic health among Malaysian adolescents: a systematic review
Source: BMC Public Health. 2019 Feb 28;19:251. doi: 10.1186/s12889-019-6557-z (PMC6396523; doi:10.1186/s12889-019-6557-z)
Supplement: Supplementary file 1 — Search terms. This table gives the search terms used for PubMed, Science Direct, Cochrane Review and Web of Science. (DOCX 29 kb) [file 12889_2019_6557_MOESM1_ESM.docx]

**Additional file 1. Search strategies**

**Databases**

The PubMed, Science Direct, Cochrane Review and Web of Science databases

**PubMed**

Combined (Diet OR Activity) AND Adolescents AND Malaysian

((((((life style [MeSH Terms] OR ("life"[tiab] AND "style"[tiab]) OR "life style"[tiab] OR "lifestyle"[tiab] OR “Diets”[tiab] OR “Diet”[tiab] OR “Nutrition”[tiab] OR “Nutritional”[tiab] OR “Nutrient”[tiab] OR “Nutrients”[tiab] OR "Food"[tiab] OR "Foods"[tiab] OR "Eating"[tiab] OR (("dietary"[tiab] OR "diet"[tiab]) AND (“Pattern”[tiab] OR “patterns”[tiab])) OR ("eating"[tiab] AND (“Pattern”[tiab] OR “patterns”[tiab])) OR (("food"[tiab] OR "foods"[tiab] OR "diet"[tiab] OR "dietary"[tiab] OR "nutrient"[tiab] OR "nutritional"[tiab] OR “calorie”[tiab] OR “energy”[tiab] OR “fat”[tiab] OR “protein”[tiab] OR “carbohydrate”[tiab] OR “fibre”[tiab] OR “fiber”[tiab] OR “kilojoule”[tiab] OR “sugar”[tiab]) AND ("intake"[tiab] OR "consumption"[tiab] OR "habit"[tiab] OR "habits"[tiab] OR "record"[tiab] OR "records"[tiab] OR "status"[tiab] OR "Assessment"[tiab] OR "survey"[tiab] OR "survey"[tiab] OR "questionnaire"[tiab] OR "questionnaires"[tiab])) OR "Macronutrients"[tiab] OR ("dietary"[tiab] AND ("fat"[tiab] OR "fats"[tiab] OR "protein"[tiab] OR "proteins"[tiab] OR carbohydrate[tiab] OR carbohydrates[tiab] OR “sugar”[tiab])) OR "dietary fats"[tiab] OR "dietary fat"[tiab] OR "dietary proteins"[tiab] OR "dietary protein"[tiab] OR "dietary carbohydrates"[tiab] OR "dietary carbohydrate"[tiab] OR "High fat" [tiab] OR "low fat"[tiab] OR "Fatty foods"[tiab] OR “Fruit”[tiab] OR “Vegetable”[tiab] OR “Fruits”[tiab] OR “Vegetables”[tiab] OR "Sugar sweetened beverages"[tiab] OR "Sugar-sweetened beverage"[tiab] OR "Carbonated drink"[tiab] OR "Carbonated beverages"[tiab] OR "Carbohydrate Loading diet"[tiab] OR "Soft drinks"[tiab] OR "Saturated fat"[tiab] OR "Processed foods"[tiab] OR "Pre-packaged foods"[tiab] OR "Fast foods"[tiab] OR "Energy dense foods"[tiab] OR "Convenience foods"[tiab] OR "Discretionary foods"[tiab] OR "Discretionary snacks"[tiab] OR “Glycemic index”[tiab] OR “Glycemic load”[tiab] OR “Dairy”[tiab] OR “Yoghurt”[tiab] OR “Milk”[tiab] OR “Cheese”[tiab] OR “Bread”[tiab] OR “Cereal”[tiab] OR “Pasta”[tiab] OR “Rice”[tiab] OR “Noodles”[tiab] OR “Beans”[tiab] OR “Legumes”[tiab] OR “Fish”[tiab] OR “Meat”[tiab] OR “Poultry”[tiab] OR “Beef”[tiab] OR “Lamb”[tiab] OR “Pork”[tiab] OR “Offal”[tiab] OR “Fried”[tiab] OR ((“Energy”[tiab] OR “calorie”[tiab]) AND (“dense”[tiab] OR “density”[tiab])) OR “Non-core food”[tiab] OR “Extra food”[tiab] OR “Breakfast”[tiab] OR “Lunch”[tiab] OR “Dinner”[tiab] OR “Snack”[tiab] OR “Drink”[tiab]))) OR ((exercise [MeSH Terms] OR "exercise"[tiab] OR ("physical"[tiab] AND ("activity"[tiab] OR "inactivity"[tiab]) OR "physical activity"[tiab] OR (physical*[tiab] AND (activ*[tiab] OR inactiv*[tiab]) OR sports[MeSH Terms] OR "sports"[tiab] OR "sport"[tiab] OR "Aerobics"[tiab] OR "Dance"[tiab] OR "Movement"[tiab] OR "Body movement"[tiab] OR "Youth Sports "[tiab] OR “Pedometer"[tiab] OR "Leisure time"[tiab] OR "Energy expenditure"[tiab] OR "Energy metabolism"[tiab] OR sedentary[tiab])))) AND ((“Adolescent”[tiab] OR “Adolescents”[tiab] OR “Adolescence”[tiab] OR "Teen"[tiab] OR "Teens"[tiab] OR "Teenage"[tiab] OR "Teenagehood"[tiab] OR "Teenager"[tiab] OR "Teenaged"[tiab] OR "Teenagers"[tiab] OR "Youth"[tiab] OR "Youths"[tiab] OR "Youthhood"[tiab] OR "Juvenile"[tiab] OR "Juveniles"[tiab] OR "Child"[tiab] OR "Childhood"[tiab] OR "Children"[tiab] OR "Boys"[tiab] OR "Girls"[tiab] OR "Female Adolescents" [tiab] OR "Male Adolescents"[tiab]))) AND (("Malaysia"[MeSH Terms] OR "Malay"[tiab] OR "Malaysian"[tiab] OR (("Malaysian”[tiab] OR “Malay”[tiab] OR “Malaysia”[tiab]) AND (“Chinese"[tiab] OR "Indian"[tiab] OR "Peninsular"[tiab])) OR "Kuala Lumpur"[tiab] OR "Penang"[tiab] OR "Selangor"[tiab] OR "Johor"[tiab] OR "Sabah"[tiab] OR "Sarawak"[tiab] OR "Malacca"[tiab] OR "Perak"[tiab] OR "Kedah"[tiab] OR "Pahang"[tiab] OR "Kelantan"[tiab ] OR "Terengganu"[tiab] OR "Negeri Sembilan"[tiab] OR "Perlis"[tiab]))

**Science Direct**

Lifestyle OR diet* OR nutrition* OR eat*) AND (exercise OR physical activit*) AND (adolescen* OR teen*) AND Malay*

**Web of Science**

TS= (life style OR lifestyle OR Diets OR Diet OR Nutrition OR Nutritional OR Nutrient OR Nutrients OR Food OR Foods OR Eating OR dietary OR diet)

AND

TS= (Pattern* OR eating AND Pattern* OR food OR foods OR diet OR dietary OR nutrient OR nutritional OR calorie OR energy OR fat OR protein OR carbohydrate OR fiber OR fiber OR kilojoule OR sugar)

AND

TS=(intake OR consumption OR habit OR habits OR (record OR records OR status OR Assessment OR survey OR survey OR questionnaire OR questionnaires) OR Macronutrients OR (dietary AND (fat OR fats OR protein OR proteins OR carbohydrate OR carbohydrates OR sugar)) OR dietary fats OR dietary fat OR dietary proteins OR dietary protein OR dietary carbohydrates OR dietary carbohydrate OR High fat OR low fat OR Fatty foods OR Fruit OR Vegetable OR Fruits OR Vegetables OR Sugar sweetened beverages OR Sugar-sweetened beverage OR Carbonated drink OR Carbonated beverages OR Carbohydrate Loading diet OR Soft drinks OR Saturated fat OR Processed foods OR Pre-packaged foods OR Fast foods OR Energy dense foods OR Convenience foods OR Discretionary foods OR Discretionary snacks OR Glycemic index OR Glycemic load OR Dairy OR Yoghurt OR Milk OR Cheese OR Bread OR Cereal OR Pasta OR Rice OR Noodles OR Beans OR Legumes OR Fish OR Meat OR Poultry OR Beef OR Lamb OR Pork OR Offal OR Fried OR Energy OR calorie)

AND

TS=(dense OR density OR Non-core food OR Extra food OR Breakfast OR Lunch OR Dinner OR Snack OR Drink OR exercise OR exercise OR physical inactivit* OR physical activit* OR sports OR sport OR Aerobics OR Dance OR Movement OR Body movement OR Youth Sports OR Pedometer OR Leisure time OR Energy expenditure OR Energy metabolism OR sedentary)

AND

TS= ((Adolescent OR Adolescents OR Adolescence OR Teen OR Teens OR Teenage OR Teenagehood OR Teenager OR Teenaged OR Teenagers OR Youth OR Youths OR Youthhood OR Juvenile OR Juveniles OR Child OR Childhood OR Children OR Boys OR Girls OR Female Adolescents OR Male Adolescents)))

AND

TS=(Malaysia OR Malay OR Malaysian OR Malaysian OR Malay OR Malaysia OR Chinese OR Indian OR Peninsular OR Kuala Lumpur OR Penang OR Selangor OR Johor OR Sabah OR Sarawak OR Malacca OR Perak OR Kedah OR Pahang OR Kelantan OR Terengganu OR Negeri Sembilan OR Perlis)

**Cochrane Review**

#1 MeSH descriptor: [Life Style] explode all trees

#2 (life:ti,ab and style:ti,ab or "life style":ti,ab or lifestyle:ti,ab or Diets:ti,ab or Diet:ti,ab or Nutrition:ti,ab or Nutritional:ti,ab or Nutrient:ti,ab or Nutrients:ti,ab or Food:ti,ab or Foods:ti,ab or Eating:ti,ab or dietary:ti,ab or diet:ti,ab)

#3 (Pattern:ti,ab or patterns:ti,ab or eating:ti,ab or food:ti,ab or foods:ti,ab or diet:ti,ab or dietary:ti,ab or nutrient:ti,ab or nutritional:ti,ab or calorie: ti,ab or energy:ti,ab or fat:ti,ab or protein: ti,ab or carbohydrate:ti,ab or fibre:ti,ab or fiber:ti,ab or kilojoule: ti,ab or sugar:ti,ab)

#4 (intake:ti,ab or consumption:ti,ab or habit:ti,ab or habits:ti,ab or record:ti,ab or records:ti,ab or status:ti,ab or Assessment:ti,ab or survey:ti,ab or survey:ti,ab or questionnaire:ti,ab or questionnaires:ti,ab or Macronutrients:ti,ab or dietary:ti,ab)

#5 (dense or density or Non-core food or Extra food or Breakfast or Lunch or Dinner or Snack or Drink or exercise or exercise or physical inactivit* or physical activit* or sports or sport or Aerobics or Dance or Movement or Body movement or Youth Sports or Pedometer or Leisure time or Energy expenditure or Energy metabolism or sedentary)

#6 (fat:ti,ab or fats: ti,ab or protein:ti,ab or proteins: ti,ab or carbohydrate:ti,ab or carbohydrates:ti,ab or sugar:ti,ab or dietary fats:ti,ab or "dietary fat":ti,ab or dietary proteins:ti,ab or dietary protein:ti,ab or dietary carbohydrates:ti,ab or dietary carbohydrate:ti,ab or High fat or low fat:ti,ab or Fatty foods:ti,ab or Fruit:ti,ab or Vegetable:ti,ab or Fruits:ti,ab or Vegetables:ti,ab or Sugar sweetened beverages: ti,ab or Sugar-sweetened beverage:ti,ab or Carbonated drink:ti,ab or Carbonated beverages: ti,ab or Carbohydrate Loading diet:ti,ab or Soft drinks:ti,ab or Saturated fat:ti,ab or Processed foods:ti,ab or Pre-packaged foods:ti,ab or Fast foods:ti,ab or Energy dense foods:ti,ab or Convenience foods:ti,ab or Discretionary foods:ti,ab or Discretionary snacks:ti,ab or Glycemic index:ti,ab or Glycemic load:ti,ab or Dairy:ti,ab or Yoghurt:ti,ab or Milk:ti,ab or Cheese:ti,ab or Bread:ti,ab or Cereal:ti,ab or Pasta:ti,ab or Rice:ti,ab or Noodles:ti,ab or Beans:ti,ab or Legumes:ti,ab or Fish:ti,ab or Meat:ti,ab or Poultry:ti,ab or Beef:ti,ab or Lamb:ti,ab or Pork:ti,ab or Offal:ti,ab or Fried:ti,ab or Energy:ti,ab or calorie:ti,ab)

#7 exercise:ti,ab or (physical activity:ti,ab or inactivity:ti,ab) or "physical activity":ti,ab or (physical*:ti,ab and (activ*:ti,ab or inactiv*:ti,ab) or "sports" or sports:ti,ab or sport:ti,ab or Aerobics:ti,ab or Dance:ti,ab or Movement:ti,ab or

"Body movement":ti,ab or "Youth Sports ":ti,ab or Pedometer:ti,ab or "Leisure time":ti,ab or "Energy expenditure":ti,ab or "Energy metabolism":ti,ab or sedentary:ti,ab)

#8 MeSH descriptor: [Exercise] explode all trees

#9 MeSH descriptor: [Sports] explode all trees

#10 #1 or #2 or #3 or #4 or #5 or #6

#11 #7, #8, or #9

#12 (Adolescent:ti,ab or Adolescents:ti,ab or Adolescence:ti,ab or Teen:ti,ab or Teens:ti,ab or Teenage:ti,ab or Teenagehood:ti,ab or Teenager:ti,ab or Teenaged:ti,ab or Teenagers:ti,ab or Youth:ti,ab or Youths:ti,ab or Youthhood:ti,ab or Juvenile:ti,ab or Juveniles:ti,ab or Child:ti,ab or Childhood:ti,ab or Children:ti,ab or Boys:ti,ab or Girls:ti,ab or Female Adolescents or Male Adolescents:ti,ab)

#13 MeSH descriptor: [Malaysia] explode all trees

#14 (Kuala Lumpur:ti,ab or Penang:ti,ab or Selangor:ti,ab or Johor:ti,ab or Sabah:ti,ab or Sarawak:ti,ab or Malacca:ti,ab or Perak:ti,ab or Kedah:ti,ab or Pahang:ti,ab or Kelantan:ti,ab or Terengganu:ti,ab or Negeri Sembilan:ti,ab or Perlis:ti,ab)

#15 (Malay: ti,ab or Malaysian:ti,ab or Malaysian:ti,ab or Malay:ti,ab or Malaysia:ti,ab)

#16 (Chinese: ti,ab or Indian:ti,ab or Peninsular:ti,ab)

#17 #13 or #14 or #15 or #16

#18 #10 and #11 and #12 and #17

#19 MeSH descriptor: [Adolescent] explode all trees

#20 #12 or #19

#21 #10 and #11 and #20 and #17
